# Athlete monitoring in handball (ATHMON HB): a survey of current practice in professional women’s and men’s handball

**DOI:** 10.1186/s13102-025-01177-4

**Published:** 2025-05-20

**Authors:** Alexander-Stephan Henze, Jakob Burger, Lynn Matits, Hannes Degenhardt, Kai Fehske, Johannes Kirsten, Sebastian Viktor Waldemar Schulz

**Affiliations:** 1 Sports and Rehabilitation Medicine, University Hospital Ulm, Leimgrubenweg 14, 89075 Ulm, Germany; 2 Division of Exercise Physiology and Metabolism, University of Bayreuth, Bayreuth, Germany; 3 Division of Clinical and Biological Psychology, Institute of Education and Psychology, Ulm University, Ulm, Germany; 4 Department of Sports Orthopaedics, Technical University of Munich, Munich, Germany; 5 Department of Orthopedic and Trauma Surgery, Johanniter Waldkrankenhaus, Bonn, Germany; 6 Department of Trauma, Hand, Plastic and Reconstructive Surgery, University Hospital, Würzburg, Germany

**Keywords:** Team sports, Athletic performance, Athletic injuries, Primary prevention, Load management, Surveys and questionnaires

## Abstract

**Background:**

Athlete monitoring is becoming increasingly important in elite team sports to optimize performance and prevent injuries and other athletic health problems. A variety of objective and subjective measures are available to monitor external and internal load, well-being, and readiness to train or compete. A comprehensive Athlete Monitoring System (AMS) should include a meaningful combination of these methods and provide analysis and graphical presentation with decision support for coaches. The selection of monitoring methods to establish an AMS is influenced not only by sport-specific considerations, but also by the resources available. Our study aimed to describe the current practice of athlete monitoring in professional handball, and to examine potential gender differences.

**Methods:**

A total of 104 women’s (*n* = 44) and men’s (*n* = 60) handball teams competing in the first two national leagues in Germany and Switzerland were invited to participate in an online survey (May-August 2024). The questionnaire used for this cross-sectional study was designed by an interdisciplinary team and consisted of a maximum of 28 questions. The Mann-Whitney U test was used to test for gender differences on the extent of athlete monitoring in the following sub-areas: Injuries and illness surveillance (IIS); external and internal load; well-being and readiness.

**Results:**

The minimum response rate was 34.6% (19 men’s teams, 17 women’s teams). An IIS was performed in 75.0% of the responding teams. A total of 19 teams (52.8%) conducted further athlete monitoring, in 84.2% of cases with a comprehensive AMS, which most commonly included player tracking (13 out of 15 teams using external load measures) and the Session-Rating of Perceived Exertion-method (11 out of 19 teams using internal load measures), and customized well-being questionnaires (10 out of 18 teams using well-being measures). Men’s teams engage in significantly more IIS (94.7% men’s teams vs. 52.9% women’s teams, *p* =.019, *δ* = –0.356), player tracking (52.6% men’s teams vs. 11.8% women’s teams, *p* =.003, *δ* = –0.415) and internal load monitoring (63.2% men’s teams vs. 29.4% women’s teams, *p* =.015, *δ* = –0.387). Notably, 88.9% of teams are interested in implementing or developing an AMS.

**Conclusions:**

Our findings indicate that the majority of handball teams already use some form of IIS, and more than half of these teams have implemented an AMS, with men’s teams using significantly more player tracking, internal load and well-being measures. The apparent high level of interest in athlete monitoring highlights the importance of data-driven approaches to managing player health and performance, although selection bias must be considered. This information is valuable for practitioners seeking to implement or expand AMS in handball.

**Supplementary Information:**

The online version contains supplementary material available at 10.1186/s13102-025-01177-4.

## Background

In the context of intensifying competition schedules in professional team sports, effective load management and recovery strategies are pivotal elements in achieving optimal performance and preventing potential athletic health problems [1–4]. In addition to systematically recording sports-related injuries, illnesses, and other athletic health problems (Injury and Illness Surveillance, IIS), athlete monitoring provides important information for the decision-making process of coaches regarding the management of training loads and recovery periods [1–6]. Recent advances in microtechnology have led to the development of player tracking methods such as inertial measurement units (IMU) or local positioning systems (LPS) to objectively measure external loads in indoor team sports such as handball [2, 6–8]. Internal load as the individual psychophysiological response to external load can be measured by objective (e.g., heart rate-based measures or biomarkers) and subjective methods (e.g., RPE methods, rating of perceived exertion) [1–8], whereas validated multidimensional questionnaires are considered the gold standard for the assessment of well-being [5]. An Athlete Monitoring System (AMS) for team sports should combine such objective and subjective measures in a multimodal approach on a software-based platform to assess, analyze, and illustrate players’ external and internal loads, well-being and readiness/preparedness to train or compete and to provide a decision support for coaches in their daily work [5–9]. The selection of methods for an AMS in team sports depends on several factors, including the distinctive sport-specific characteristics, such as the environment (outdoor/indoor) and the physical demands of the various playing positions. Furthermore, the financial resources available and the associated availability of technical equipment and qualified personnel are of paramount importance [8, 9].

Indoor handball, also referred to as team handball, is regarded as one of the fastest and most physically demanding team sports globally [10, 11]. Elite handball players in Europe currently participate in up to 80 games per season underscoring the growing interest in athlete monitoring [11]. The implementation of an LPS with ultra-wideband (UWB) technology in the top German men’s league in the 2019/20 season has contributed to this trend [11]. However, it remains unclear whether German handball clubs employ this technology as a component of their AMS, or whether any objective or subjective monitoring procedures are used at all. Similarly, it is uncertain whether comparable practices are used in other professional men’s handball leagues or in women’s handball. Although recent surveys have investigated current athlete monitoring practices in various high-performance sports, no studies have yet focused on team handball [12–15]. Consequently, to the best of our knowledge, no study has examined the current use of different athlete monitoring methods in this sport.

Therefore, the main objective of our study is to provide a comprehensive overview of current athlete monitoring practices in the first two national leagues of professional women’s and men’s team handball in Germany and Switzerland. The following sub-areas will be presented: injury and illness surveillance; external and internal load; well-being and readiness to train/play. The second objective is to examine the potential gender differences on the extent of the use of monitoring methods. Based on the financial resources available to the teams, it was hypothesized that the men’s teams engage in more comprehensive athlete monitoring in the various sub-areas than the women’s teams.

## Methods

### Development of the questionnaire

The questionnaire used in this cross-sectional study was designed by an interdisciplinary team, consisting of a sports medicine specialist, who is also a team physician in the first German national handball league, a sports scientist, physiotherapist and strength and conditioning coach, and a psychologist. The approach and findings from previous studies that included surveys on the current practice of athlete monitoring in high-performance sports, other literature on athlete monitoring in team sports, and personal experience were considered [12–14]. The questionnaire was reviewed for internal consistency by two board members of *Handballärzte Deutschland e.V.*, a non-profit organization of team physicians and physiotherapists of German professional and semi-professional handball clubs. The online version was then pre-tested by four suitable individuals from the target group of this survey (two handball coaches from semi-professional teams, two sports scientists) to assess its feasibility and comprehensibility. The final version contained a maximum of 28 questions, of which 14 were single-choice and 12 were multiple-choice. It also included a prioritization question where participants ranked their athlete monitoring objectives from 1 to 4 based on importance and a 5-point Likert-type scale question, ranging from 0 to 100%, that assessed the extent to which coaches use athlete monitoring information in load management decisions (see Supplementary Material). The questionnaire was divided into the following sections: demographics (question 1 to 4); IIS (question 5 and 6); AMS purpose and platform (question 7 to 13); monitoring of external (question 14 and 15) and internal load (question 16 and 17); monitoring of fatigue and recovery, well-being, and readiness to train/play (question 18 to 25); individualization (question 26); current value and future perspectives of athlete monitoring (question 27 and 28).

### Study population and study period

The study population consisted of staff or management members of 104 handball teams playing in the first and second women’s and men’s handball leagues in Germany and Switzerland. The study was performed in accordance with the latest version of the Helsinki Declaration and was approved by the ethics committee of Ulm University (no. 138/24).

The online survey was conducted using SoSci Survey and made available to respondents at https://www.soscisurvey.de [16]. For this purpose, an email with the corresponding link was sent via the league’s offices to the 104 handball teams. Both this e-mail and the survey landing page provided detailed information about the purpose, content, scope and planned open access publication of the results, as well as the option to end participation without giving a reason. In addition, the complete anonymity of the survey, which was conducted without IP tracking and in which the answers did not allow any conclusions to be drawn about the respondent or the handball team, was explicitly mentioned. Informed consent to participate in the study was then given digitally by continuing the survey. The survey was open for four months (May 1 to August 31, 2024), with no reminder email was sent. Participants were excluded from further analysis if they did not complete the entire survey (*n* = 9) or did not report about a handball team of the study population (*n* = 1, working a junior elite team director for a national handball federation).

### Statistical analysis

Survey data were imported into JASP (Intel version 0.18.1, JASP Team 2023) for further analysis. Descriptive data are presented as raw data with absolute and relative frequencies for nominal variables and medians and quantiles for ordinal variables. The minimum response rate was calculated using the latest version of the American Association of Public Opinion Research (AAPOR) standard definitions [17]. The Mann-Whitney *U* test was used to test for differences between women’s and men’s teams, with the alternative hypothesis that men’s teams practice a more extensive athlete monitoring in each sub-area. Therefore, ordinal variables were employed to quantify the extent of each monitoring sub-area, considering the number and complexity (e.g., simple counting methods < player tracking) of the measures and the frequency with which they were used. Effect sizes were calculated as Cliff’s delta (*δ*) with 95% confidence intervals (CIs) and categorized according to Cohen as small 0.0125 ≤ X < 0.304, medium 0.304 ≤ X < 0.465, and large > 0.465 [18]. An *α* level of 0.05 (one-tailed) was considered significant.

## Results

### Study population

A total of 36 out of the invited 104 handball teams completed the entire survey, yielding a minimum response rate of 34.6%. Table 1 presents the national league affiliation and participation in a European club competition of the responding handball teams.

**Table 1:** National league affiliation/participation in a European handball federation (EHF) competition of the handball teams participating in the ATHMON HB (Athlete monitoring in handball) online survey

	Overall	1st national league	2nd national league	EHF champions league	EHF European league	EHF European cup
Women	17	10	7	0	2	3
Men	19	14	5	1	3	1
Sum	36	24	12	1	5	4

**Fig. 1:**
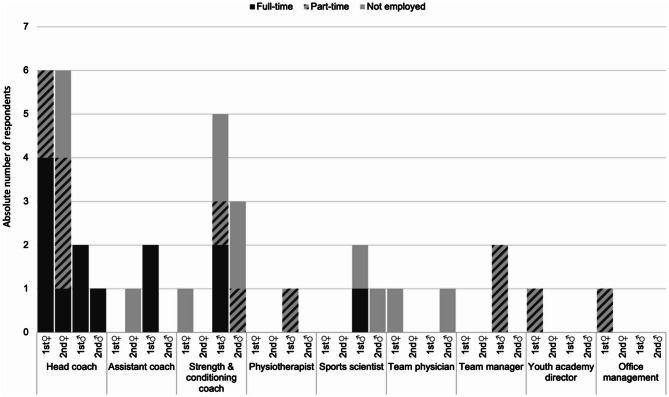
League-specific distribution of responding team representatives, professional roles and employment type within the handball club. The different roles of the responding team representatives are sorted by league affiliation (♀ symbol: women’s leagues; ♂ symbol: men’s leagues) on the X-axis. The absolute number of responding team representatives is plotted on the Y-axis, with color and pattern indicating the extent of employment in the handball club (see legend). *N* = 36

Most respondents were head coaches (*n* = 15, 80% of women’s teams) or strength and conditioning coaches (*n* = 9, 89% of men’s teams). Figure 1 provides an overview of their league-specific distribution, professional roles and type of employment.

### Injury and illness surveillance

75.0% of responding handball teams exceed an IIS (*n* = 27). A substantial proportion of teams (61.1%, *n* = 22) monitor acute and overuse injuries and other athletic health problems like illness, non-functional overreaching, or overtraining syndrome. Of the nine respondents who do not conduct an IIS, eight represent women’s teams. Significant gender differences were observed, indicating that men’s teams were more likely to perform a more extensive IIS, *U* = 104.000, *p* =.019, *δ* = –0.356 [–∞; –0.051]. In most cases (74.1%, *n* = 20), IIS were performed by multiple staff members, typically physiotherapists (74.1%), team physicians (48.1%), and strength and conditioning coaches (44.1%).

### Athlete monitoring systems

Of the teams conducting IIS, 63% (*n* = 17; women: 44.4%; men: 72.2%) reported regularly monitoring their athletes beyond injuries and other health problems. In addition, athlete monitoring is carried out for two handball teams without performing an IIS (*n* = 2).

When asked to rank the purposes of athlete monitoring (prevention of athletic health problems; improvement/maintenance of performance; evaluation of effectiveness of training/prevention programs; evaluation of effectiveness of recovery methods) in order of importance, most respondents ranked ‘prevention of athletic health problems’ as the most important purpose (68.4%) and ‘improvement/maintenance of performance’ as the second most important reason (57.9%).

**Fig. 2:**
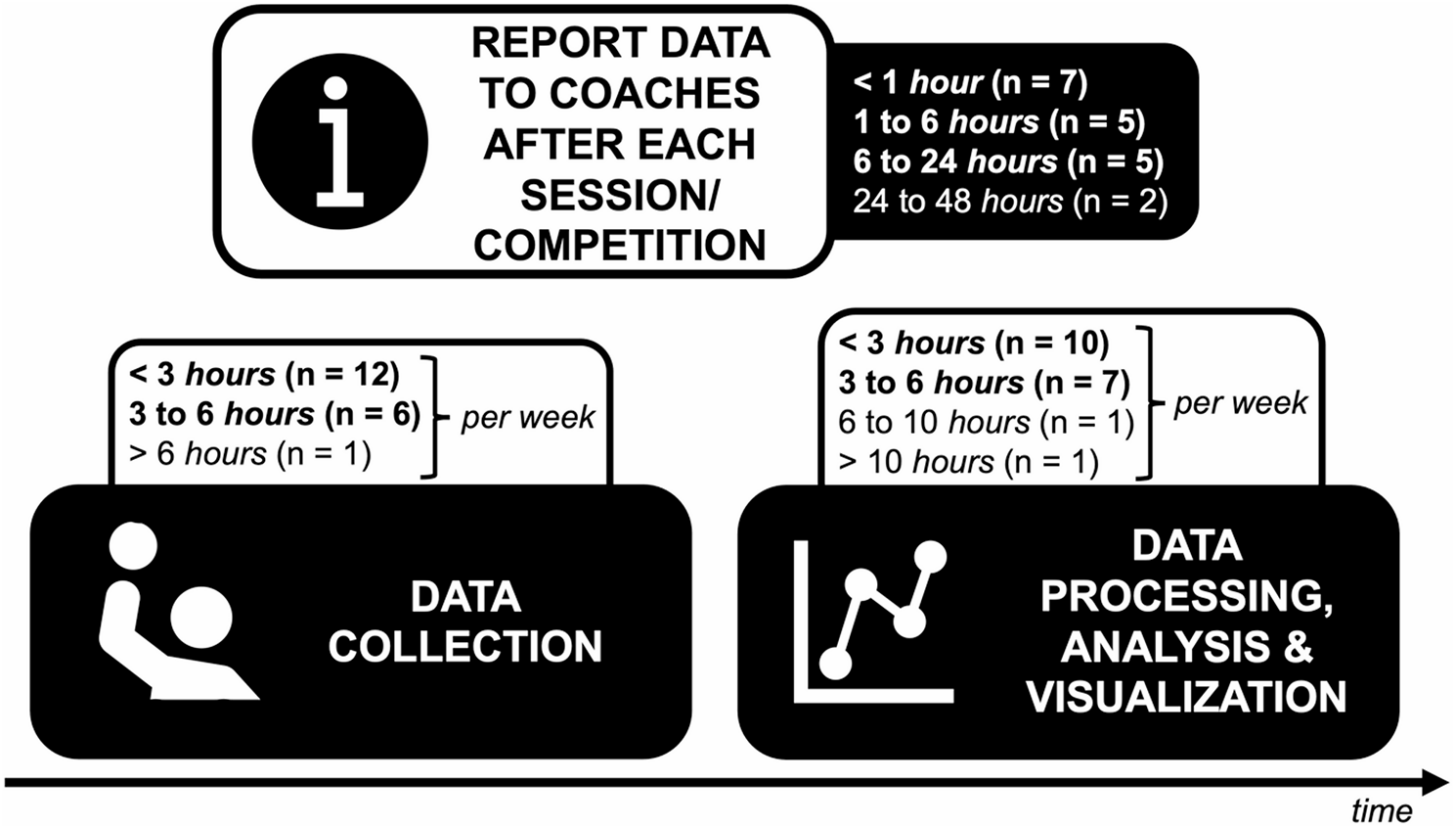
Timeframe for each step of athlete monitoring in professional handball. The time specified for data collection and processing, analysis and graphical visualization refers to the weekly working hours of the staff members. The time required to inform the coaches about the results of the various monitoring methods pertains to each training or competition session

Most teams performing further athlete monitoring utilize comprehensive AMSs (84.2%, *n* = 16). Among these, 87.5% (*n* = 14) use a commercial AMS software application (open-source or customized: *n* = 1 each). Notably, 70.6% of these AMSs include a decision-support system. Only three out of 19 teams rely solely on simple spreadsheets for data collection, processing, and analysis. Typically, staff spend up to six hours per week on data collection and up to ten hours per week on processing and analysis. Usually, the analyzed and graphical visualized data is delivered to coaches within 24 h (see Fig. 2).

### Load monitoring

**Fig. 3:**
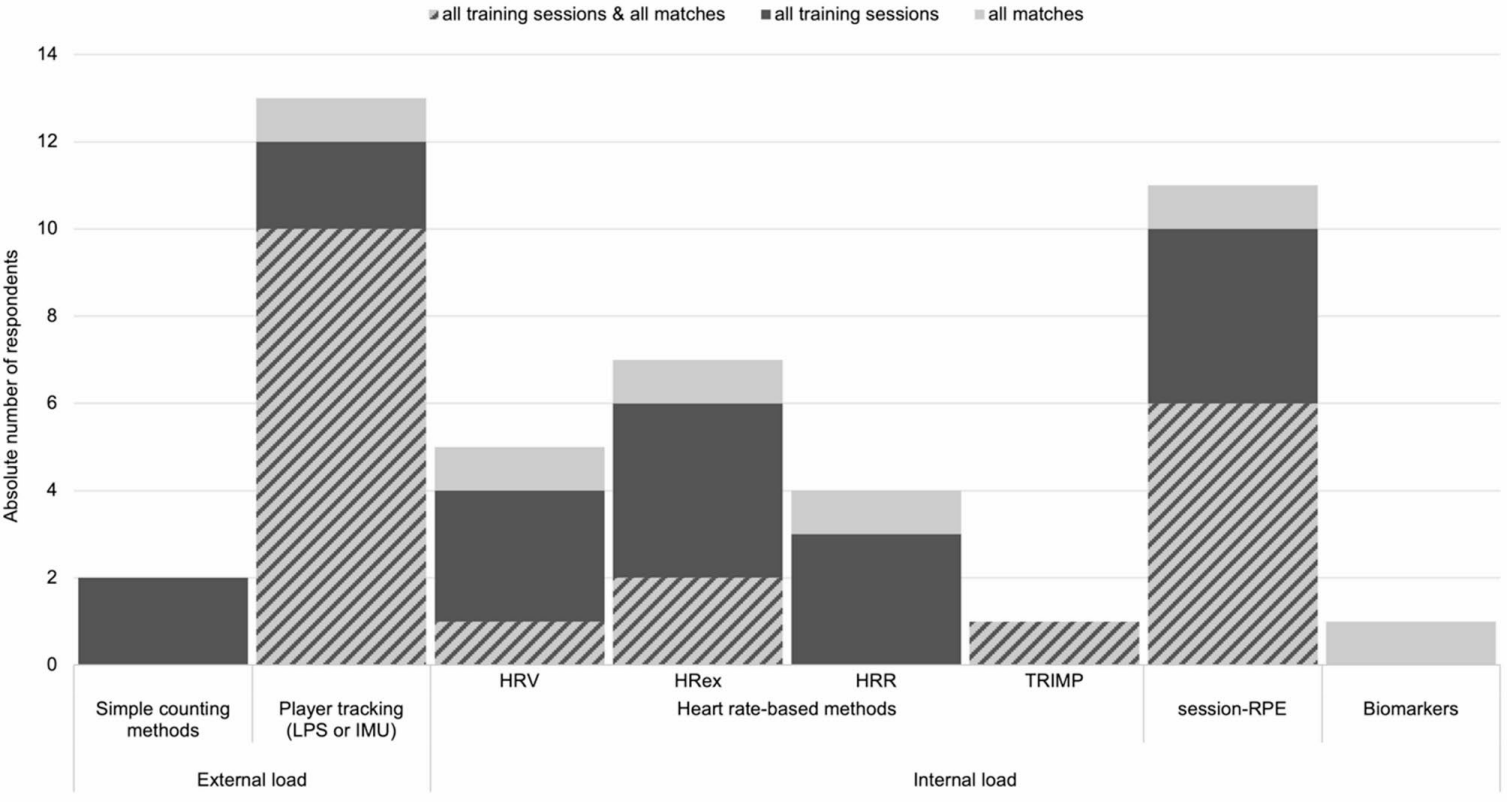
Methods used in professional team handball to monitor the external and internal load of players. The different monitoring methods are sorted on the X-axis, with color and pattern indicating the frequency of use (see legend). The absolute number of responding team representatives is plotted on the Y-axis, *n* = 19. Abbreviations: LPS Local positioning system, IMU Inertial measurement unit, HRV Heart rate variability, HR_ex_ Heart rate exercise, HRR Heart rate recovery, TRIMP Training impulse, RPE Rating of perceived exertion

A total of 93.8% of teams using an AMS monitor the players’ external load (*n* = 15). For this purpose, 76.9% use an LPS (see Fig. 3). One men’s first-league team tracks players with IMUs in every training session or competition, two women’s second-league teams use simple counting methods. Significant gender differences were observed, indicating that men’s teams were more likely to use more extensive external load monitoring, *U* = 94.500, *p* =.003, *δ* = –0.415 [–∞; –0.119].

All teams monitoring athletes have included internal load measures (see Fig. 3), specifically session-RPE (70.6%) and heart rate (HR)-based methods (58.8%). Significant gender differences were observed, indicating that men’s teams were more likely to use more extensive internal load monitoring, *U* = 99.000, *p* =.015, *δ* = –0.387 [–∞; –0.087].

### Well-being and readiness

**Fig. 4:**
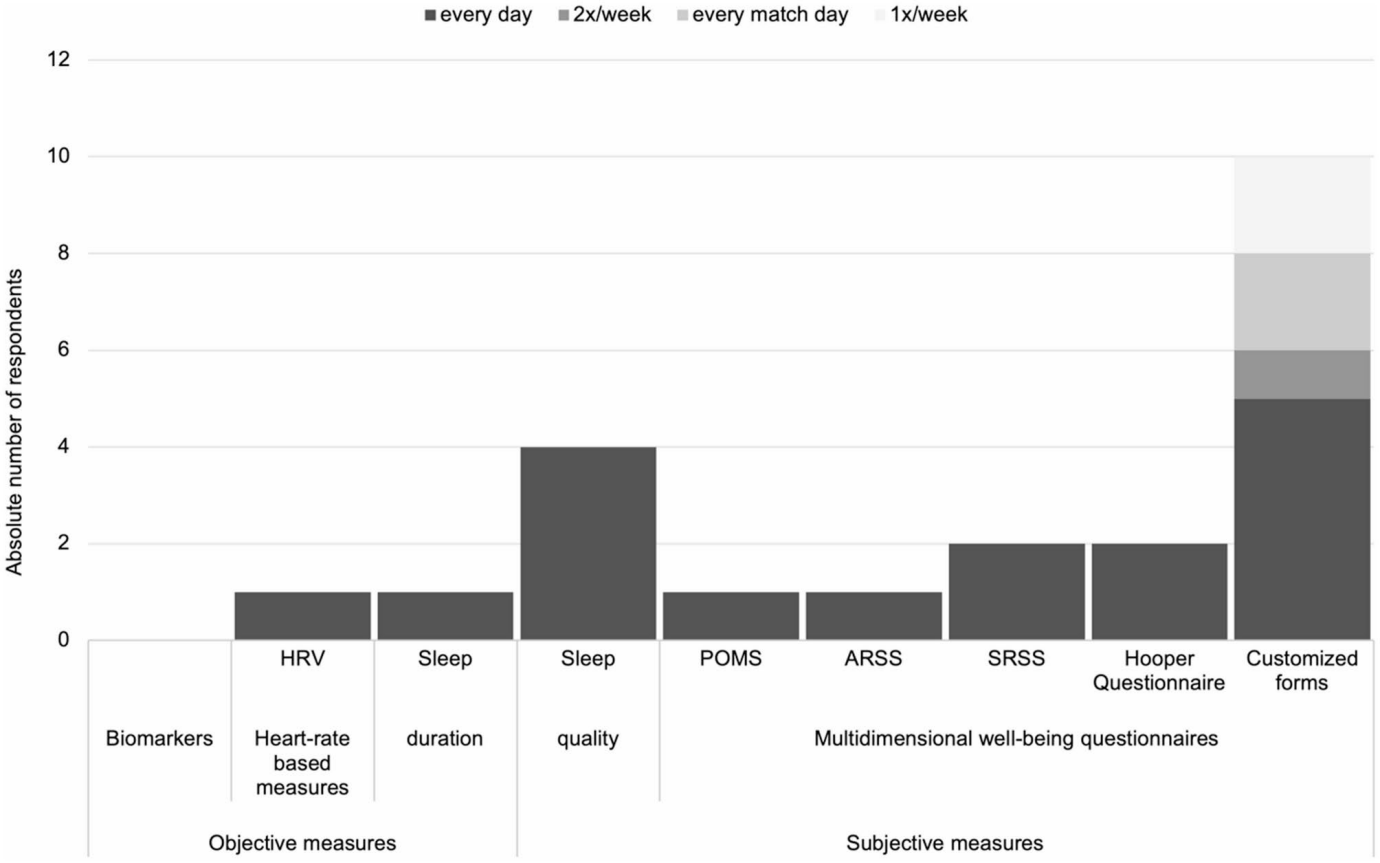
Methods used in professional handball to monitor perceptual well-being. The different monitoring methods are sorted on the X-axis, with color and pattern indicating the frequency of use (see legend). The absolute number of responding team representatives is plotted on the Y-axis, *n* = 18. Abbreviations: HRV Heart rate variability, POMS Profile of Mood States, ARSS Acute Recovery and Stress Scale, SRSS Short Recovery and Stress Scale

No team indicated to use biomarkers for well-being monitoring. However, most teams performing athlete monitoring (*n* = 18) assess well-being with athlete self-report measures (ASRMs), in over two-thirds of cases on a daily basis. In addition to validated questionnaires such as the *Profile of Mood States* (POMS) or the sport-specific *Short Recovery and Stress Scale* (SRSS), custom-designed questionnaires are used by over half of the teams (*n* = 10) utilizing ASRMs for the assessment of players’ well-being (see Fig. 4). There was no gender difference observed on the extent of well-being monitoring (*U* = 142.000, *p* =.24).

**Fig. 5:**
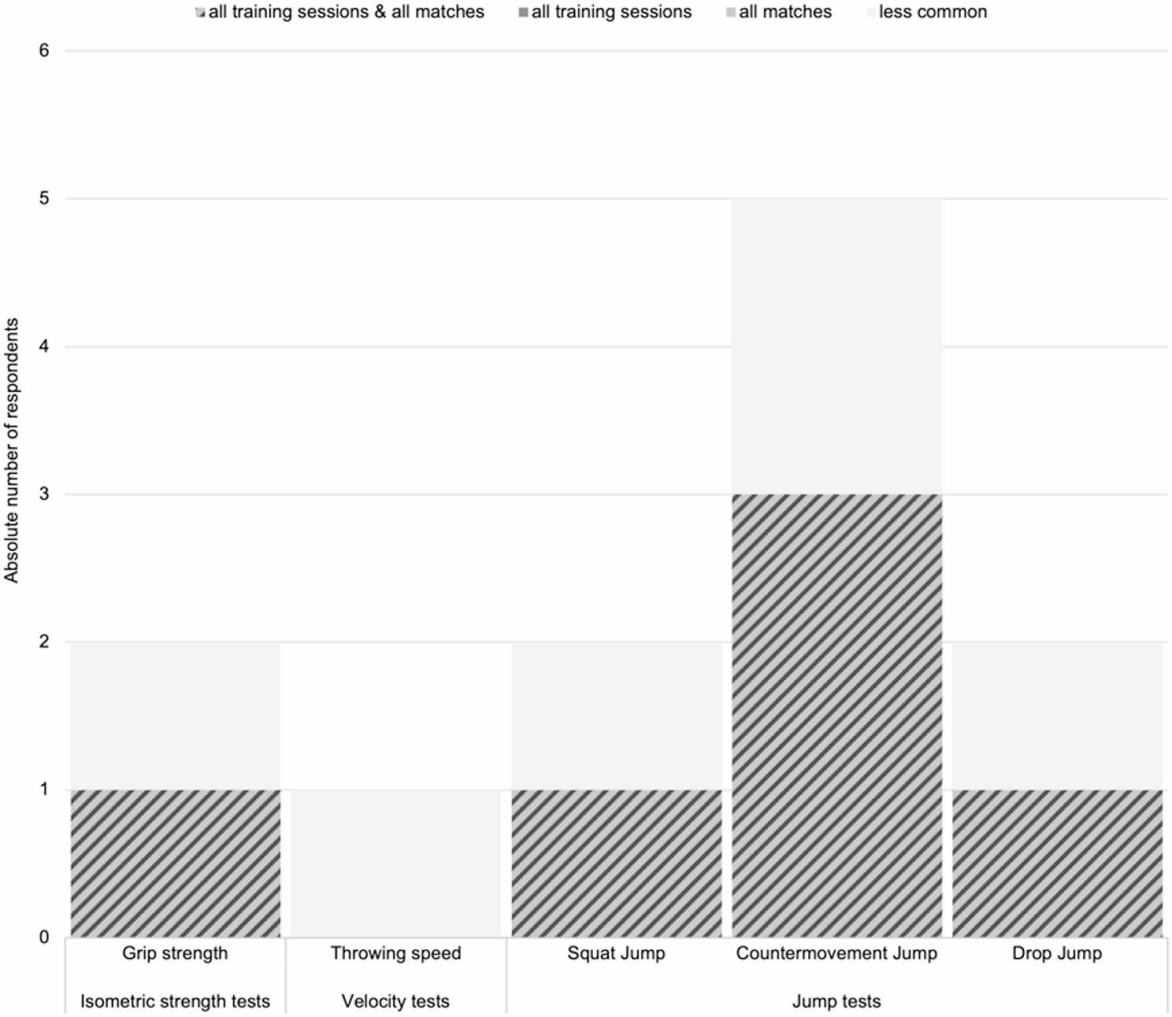
Methods used in professional handball to monitor readiness to train/play. The different monitoring methods are sorted on the X-axis, with color and pattern indicating the frequency of use (see legend). The absolute number of responding team representatives is plotted on the Y-axis, *n* = 5

Only five out of the 36 respondents indicated that their teams perform regular motor testing to assess readiness to train/play (see Fig. 5). Due to the small sample size, no interference statistics method was applied to this sub-area.

### Individualization

In response to the inquiry regarding the implementation of individualized monitoring strategies, only three men’s first league teams provided comprehensive information. Two teams employ the use of Z-scores, while one team utilizes a Bayesian approach to implement individualization.

### Current value and future perspectives

Most respondents (*n* = 14) indicated that athlete monitoring results are used by coaches to a degree of 50–75% (*n* = 7 each) for decision-making in daily load management. 88.9% of responding teams, including those not currently monitoring athletes, expressed a strong interest in implementing at least one monitoring method. Of the teams that do not currently perform any form of athlete monitoring, 73.3% prefer to implement a comprehensive AMS. All women’s teams that monitor their athletes would like to use at least one additional method. In contrast, only two-thirds of men’s teams would like to expand their AMS.

## Discussion

The main objective of this observational study was to assess the current practice for monitoring professional handball players in the context of load and recovery management. The present study is the first to describe the current practice of athlete monitoring in team handball and in any form of handball in general, providing detailed information on the largest number of teams from a single team sport to date. Our findings indicate that a substantial proportion of professional handball teams perform an IIS and further athlete monitoring, usually utilizing a comprehensive AMS with specialized commercial software applications. These AMSs typically contain LPS or IMU player tracking and the session-RPE method for load monitoring, and customized ASRMs for well-being assessments.

The second objective of our study was to examine potential gender differences in the extent of monitoring methods used in the various sub-areas. Our results confirmed our hypothesis that men’s teams engage in significantly more extensive IIS and load monitoring than women’s teams, with a medium strong effect observed for both.

### Injury and illness surveillance

Systematic IIS is a basic tool that, in addition to individual performance indicators, provides health-related outcome measures to assess the effectiveness of prevention interventions or adjustments in load and recovery management [19]. Encouragingly, 94.7% of the responding men’s teams and 52.9% of women’s teams systematically record at least acute injuries. The anticipated and statistically significant differences between women’s and men’s teams might be attributed to differences in financial resources and, consequently, human resources available, including coaches and medical staff. Physiotherapists and strength and conditioning coaches, who are often salaried professionals, predominantly manage IIS in these teams.

The IIS section of our survey incorporated the current recommendations of the International Olympic Committee (IOC) Consensus Statement, which also defines the terms ‘injury’ and ‘illness’ [19]. However, for the purpose of this study, the IOC injury term has been deliberately subdivided into ‘acute injuries’ and ‘overuse injuries’. The rationale underlying this subdivision is that the documentation of acute injuries, particularly those that result in a loss of participation in team practice and competition (= time-loss injuries), is considered the minimum documentation necessary for injury monitoring purposes [19]. In Germany, the social accident insurance for professional team sport athletes (Verwaltungs-Berufsgenossenschaft, VBG) has been publishing an annual report since 2016 on injury data in the two highest men’s leagues in soccer (football), basketball, handball, and ice hockey. The ‘VBG-Sportreport’ employed also a “narrow” injury term, defining an injury “as any event in training or competition that results in either medical treatment costs or the player’s inability to participate in practice or match-play” [20]. This term excludes “non-post-traumatic chronic damage” [20]. This approach of dividing acute and overuse injuries should be considered when implementing an IIS in team sports, to be able to derive more specific recommendations for prevention strategies from the information obtained. Moreover, the implementation of standardized definitions for various athletic health problems would facilitate enhanced comparisons within and across leagues, as well as between different team sports. The VBG report draws exactly this comparison and shows that handball currently has the highest cumulative seasonal injury incidence (2.3 injuries per season) and the highest average time-loss due to injury per player per season (34 days) [20]. These findings underscore the need for meticulous IIS, which facilitates the implementation of targeted prevention strategies and enables the measurement of their efficacy.

### Athlete monitoring systems

Few studies have examined the current use of athlete monitoring in high-performance sports. The first study was published in 2012, which surveyed 100 elite/non-professional and professional sports practitioners in Australia and New Zealand, encompassing various collision (*n* = 59) and non-collision (*n* = 15) team sports. This study reported a minimum response rate of 55%, with 91% of respondents indicating the use of at least one athlete monitoring method [12]. In contrast, a subsequent online survey among elite, pre-elite, and professional sports practitioners from the same region and similar team sports, had a minimum response rate of only 9.8% (52 out of 530) [13]. Notably, all of the respondents in this study used at least one monitoring method [13]. More recently, another study surveyed elite sports practitioners in the United Kingdom, including hockey and rugby 7’s, with a minimum response rate of 40% (30 out of 75) and found that 83% used an AMS [14]. However, none of these studies, nor any other to date, provided information on current practice in handball [12–15]. In our study, 52.8% of the responding handball teams indicated that they use some form of athlete monitoring. However, a certain degree of selection bias must be assumed, as teams that carry out athlete monitoring, or are at least interested in doing so, are more likely to respond to such surveys. If we assume the “minimum” in our survey that all teams that did not respond do not monitor their players (41 men’s teams and 27 women’s teams), then at least 18.3% of all teams in the surveyed sample would use some form of athlete monitoring (21.7% of men’s teams and 13.6% of women’s teams. These data show that the use of AMS in handball is still limited when compared to data from other high-performance sports [12–15].

The main purposes of athlete monitoring in handball were similar to those reported in previous surveys in other high-performance sports [12–14], with “prevention of athlete health problems” being the most important, followed by “improvement/maintenance of performance”.

In addition to our survey, only Taylor et al. reported data about the time spent collecting and analyzing monitoring data. They reported that 70% spent less than four hours per week on data collection (present study: 63.2% less than three hours per week) and 75% spent less than six hours per week on data analysis (present study: 89.5%) [12]. Therefore, it is essential to schedule this time when planning to implement an AMS. The time required to provide feedback to the coaches based on monitoring data (89.5% within 24 h) is also in line Taylor et al. (92% within 24 h) [12].

### Load monitoring

While GPS-based tracking systems are inaccurate in indoor environments, the advent of LPS technology can be seen as a ‘game changer’ in assessing physical demands and load monitoring in indoor team sports [13, 21–24]. Thirteen handball teams reported integrating LPS or IMU derived player tracking data into their AMS. Our study did not include information on which external load measures (i.e., total distance covered, speed data, metrics such as metabolic power or Player Load^®^) are used in detail. While an average of five different external load parameters are used in other sports [13], this question should be considered in future handball research. In addition, future studies should focus on typical measurement errors of these external load parameters.

Session-RPE was the most commonly used internal load monitoring tool in our study. This combined method using a single-item ASRM, and session duration is valued for its simplicity, validity, and reliability in various sports [12, 25].

As reported in the present study and in previous research [13], submaximal HR was the most commonly used HR measure, consistent with recent recommendations [26]. These recommendations emphasize that, within a multimodal monitoring approach, exercise-related HR measures appear to provide valuable objective insights into internal load [26].

### Well-being and readiness

The number of teams using ASRMs in their AMS to assess the athletes’ well-being in our study (83.3%, *n* = 15) is comparable to some previous studies (84% [12], 96% [14]). Only McGuigan et al. reported a lower number of 36% [13]. Our findings underscore the predominance of ASRMs (including RPE measures and sleep quality assessments) as the most prevalent monitoring methods [5, 12, 14, 15, 26, 27]. Validated, multidimensional ASRMs are considered the gold standard for monitoring the athletes’ well-being [5, 27]. However, the trend in sports practice shifts towards the use of customized questionnaires comprising a limited number of Likert-type items [12–15]. This approach is substantiated by the present study, which revealed that among 18 teams engaged in well-being monitoring, only four teams reported utilizing validated questionnaires like the SRSS [28, 29], while 10 teams employ customized questionnaires.

Contrary to previous studies, motor tests may play a minor role for (daily) monitoring purposes in handball. When asked about the use of motor tests to assess player readiness, five respondents indicated that they use the Countermovement Jump (CMJ), a method that is commonly used in both scientific and practical applications to monitor neuromuscular readiness and, more broadly, assess performance [6, 13, 30].

In our study, one team reported using biomarkers to monitor internal load, whereas no team reported to use biomarkers to further assess fatigue and recovery processes. However, previous research highlights the potential of creatine kinase (CK) monitoring in professional handball, with improved accuracy for classification of recovery status when using an individualized Bayesian approach [31, 32]. In addition, significant correlations were observed with SRSS items related to musculoskeletal demands [33]. As well-established biomarker in team sports [31, 34–36], CK presents a viable option for elite handball teams to integrate an objective marker into their multimodal AMS. Nevertheless, its application requires careful considerations of its weaknesses, particular inter-assay and intra-assay as well as inter-individual and intra-individual variability, to ensure reliable and usable insights [33–36].

### Strengths and limitations

The main limitation of this study is the modest sample size, which is also due to the fact that we deliberately avoided the usual procedures to increase the response rate, such as alternative forms of contact (e.g., social media) and reminder emails [12–14]. Moreover, the survey period was strategically scheduled between two regular seasons. The underlying rationale of this approach was to minimize disruption to the staff’s pre-season activities. However, our study has provided information on the use of athlete monitoring systems over the largest number of teams in a single team sport to date and, in particular, an almost equal number of women’s teams. Furthermore, in contrast to previous studies that used online surveys to examine the current use of athlete monitoring practices in high performance sports, the present study accounted for selection bias. This bias is based on our assumption that teams that do not monitor athletes are less likely to participate in the survey. In addition, it can be assumed that the teams that participated in the survey also have a greater interest in athlete monitoring. This bias must be considered when evaluating the question of interest in athlete monitoring methods.

### Practical applications

In combination with the current evidence [5, 21–23, 25, 28–30] and expert recommendations [1–4, 7, 8, 27, 36] concerning athlete monitoring in team sports, as well as the findings from previous observational studies investigating monitoring methods in handball players [32, 33, 37–40], the findings of our study have the potential to serve as a guide for practitioners when planning to introduce or further develop an AMS in handball settings. The combination of validated subjective measures to assess internal load (e.g., the session-RPE method) and well-being (e.g., multidimensional questionnaires) appears to provide a relatively simple and inexpensive basis for a multimodal AMS. In this context, an important aspect should be to replace customized ASRMs, which are very popular in practice but not validated, with appropriately short, sport-specific and validated instruments such as the SRSS. Once more resources are available, player tracking could be used as an objective method to improve AMS, although future research is needed to identify the most important parameters and metrics to assess external load in handball.

## Conclusions

The present study presents the first comprehensive overview of athlete monitoring practices in women’s and men’s team handball, providing deeper insights into injury and illness surveillance, load monitoring, and well-being assessments. The findings of our study may guide practitioners in implementing or expanding an athlete monitoring system, an area of high interest in professional handball. Future studies should investigate the external load parameters used in detail, and evaluate the potential of sport-specific, validated questionnaires to replace customized athlete self-report measures for well-being assessments.

## Electronic supplementary material

Below is the link to the electronic supplementary material.

Supplementary Material 1

## Data Availability

The datasets generated and/or analyzed in the current study are not publicly available due to the anonymity of the online survey but are available from the corresponding author on reasonable request.

## Abbreviations

AAPOR: American Association of Public Opinion Researc
AMS: Athlete Monitoring System
ASRM: Athlete Self-Report Measure
CI: Confidence Interval
HR: Heart Rate
IIS: Injury and Illness Surveillance
IMU: Inertial Measurement Unit
LPS: Local Positioning System
POMS: Profile of Mood States
RPE: Rating of Perceived Exertion
SRSS: Short Recovery and Stress Scale
UWB: Ultra-Wideband
VBG: Verwaltungs-Berufsgenossenschaft
