# Supplementary figures and images for: Athlete monitoring in handball (ATHMON HB): a survey of current practice in professional women’s and men’s handball
Source: BMC Sports Sci Med Rehabil. 2025 May 20;17:126. doi: 10.1186/s13102-025-01177-4 (PMC12090567; doi:10.1186/s13102-025-01177-4)

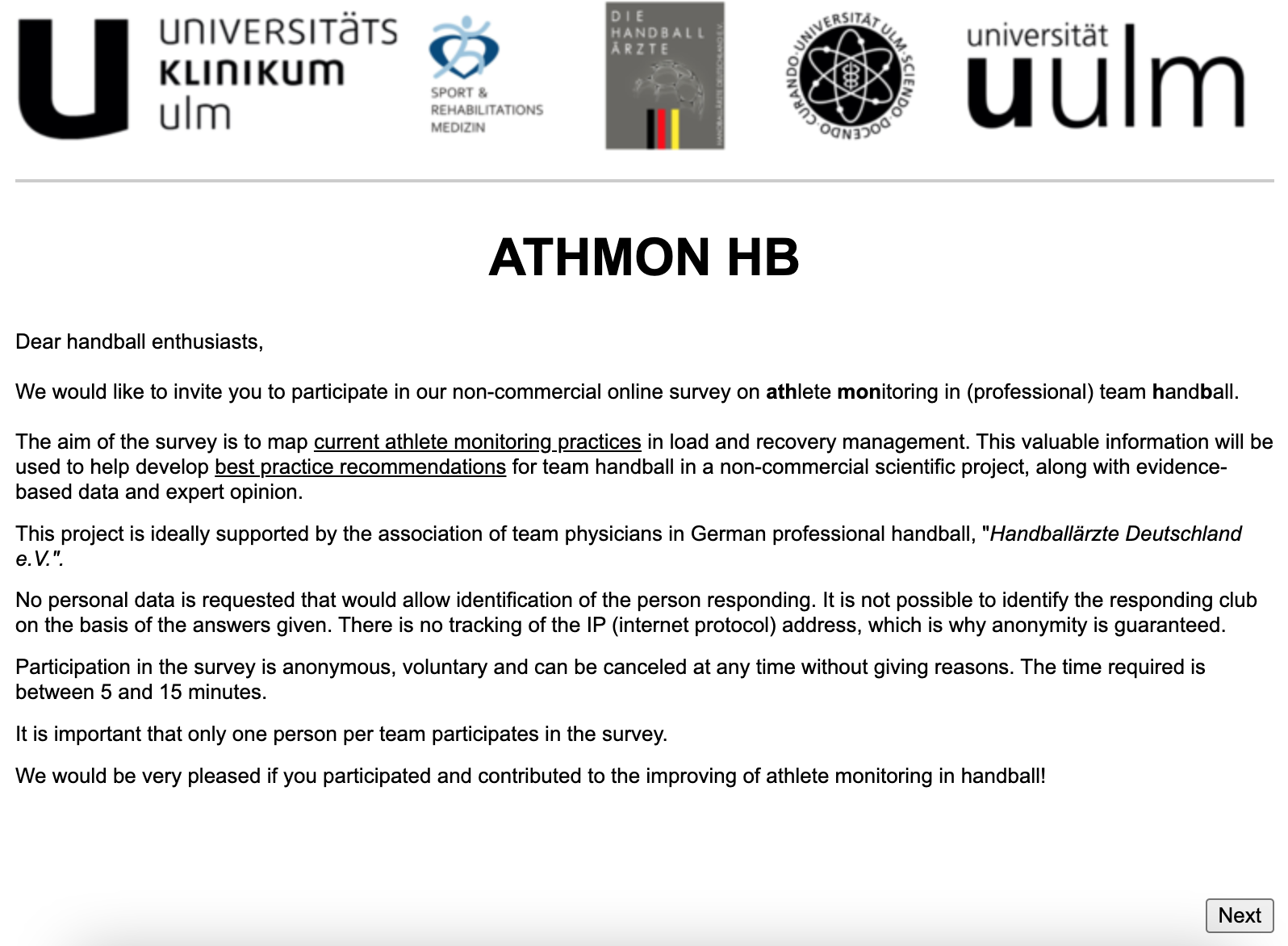

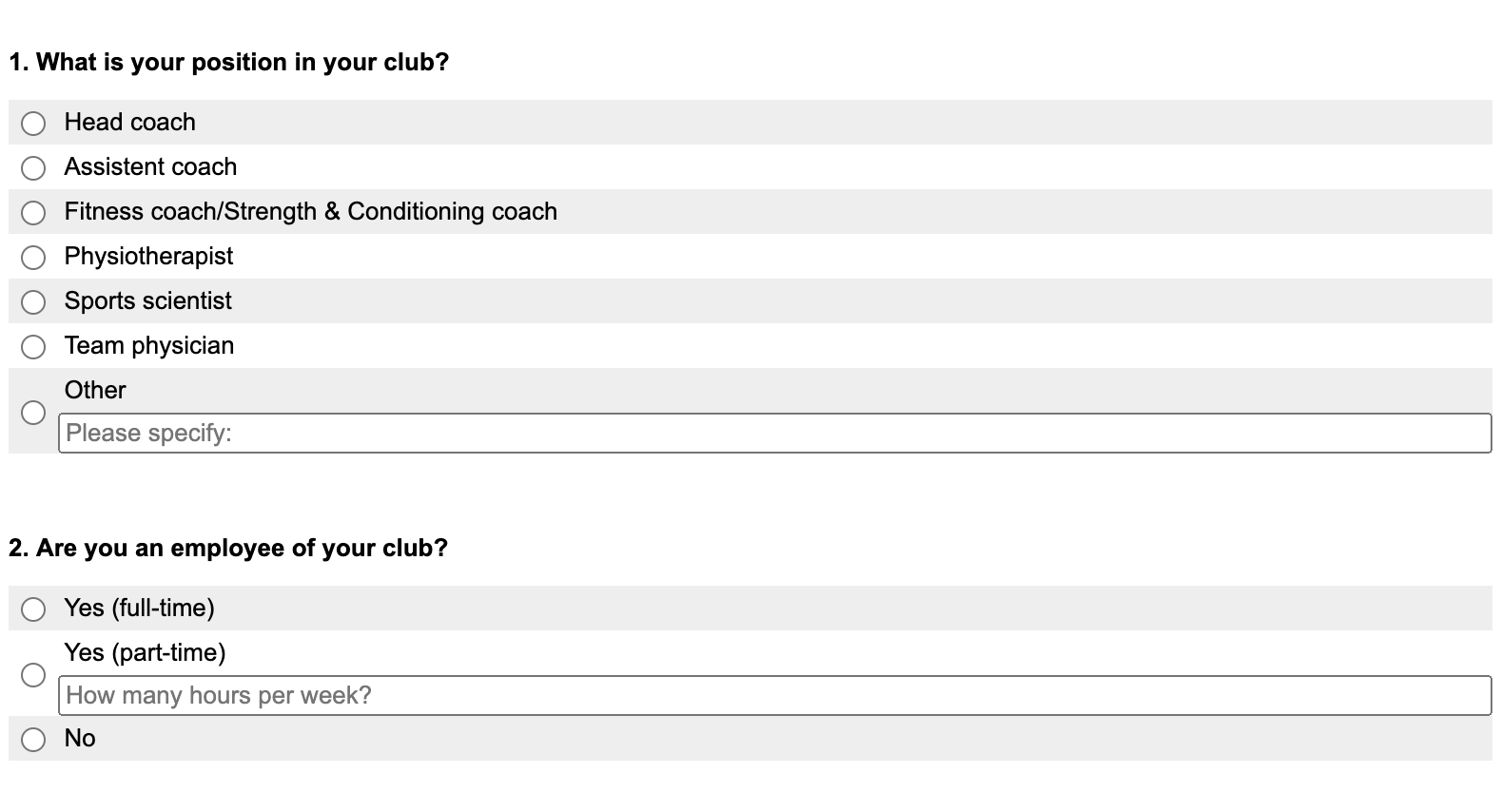

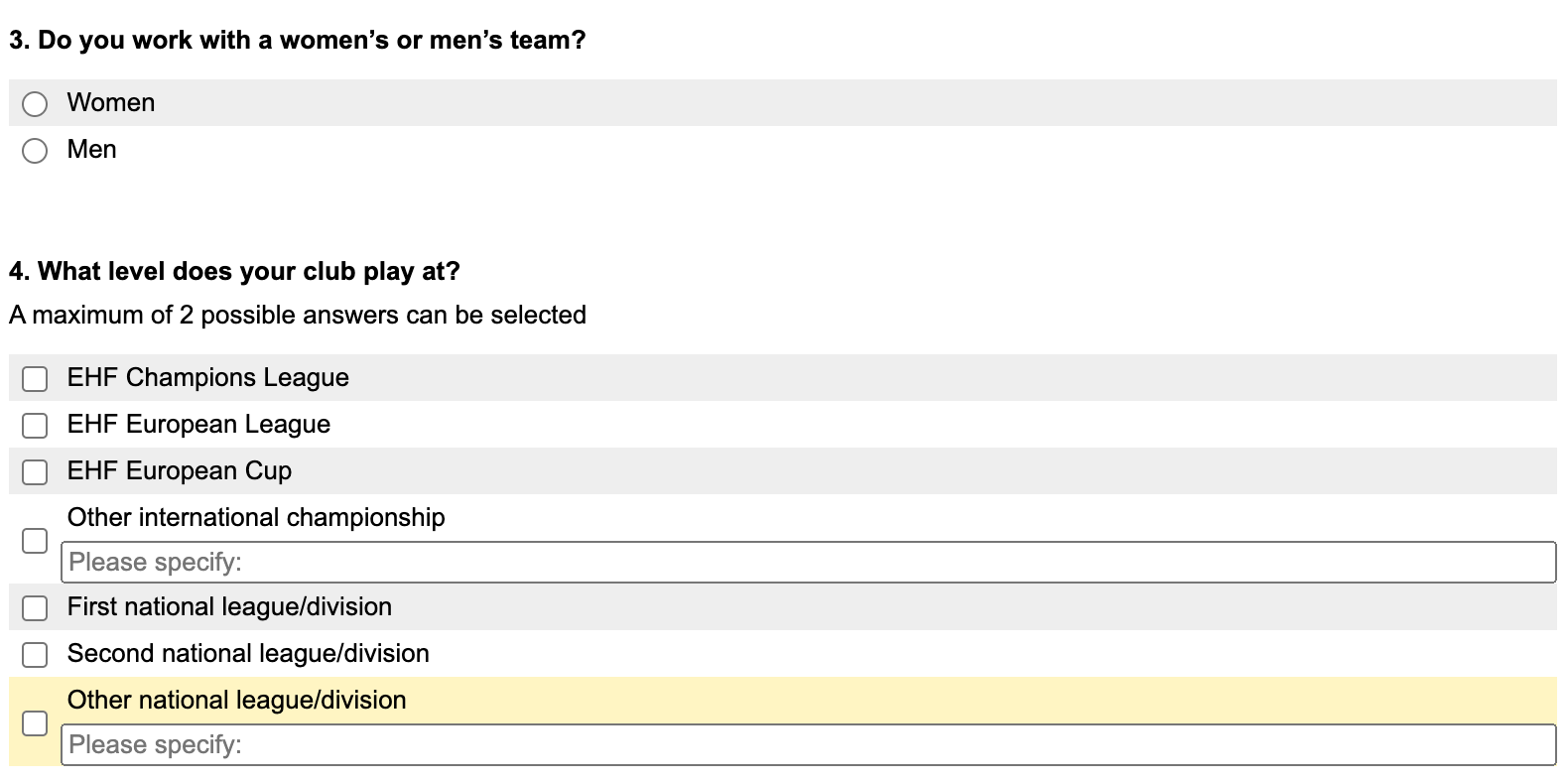


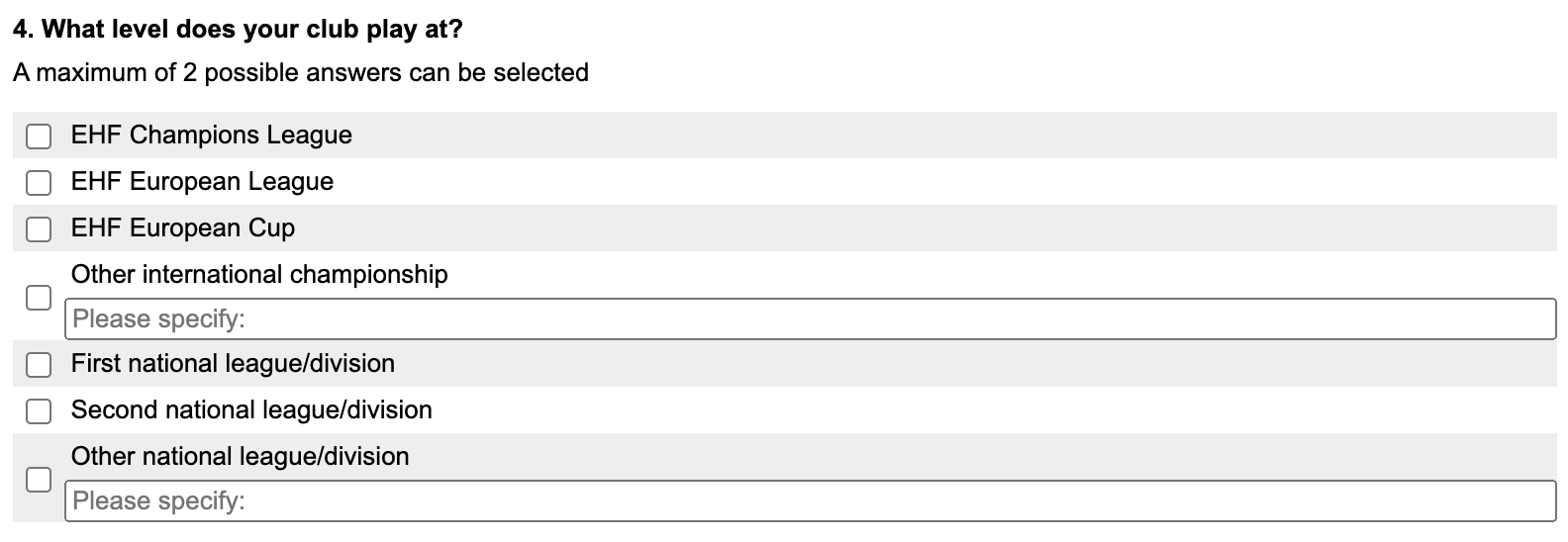

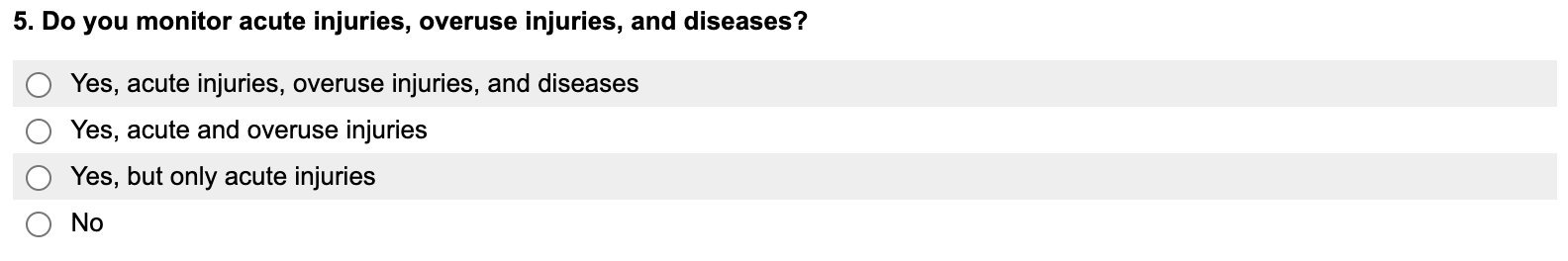

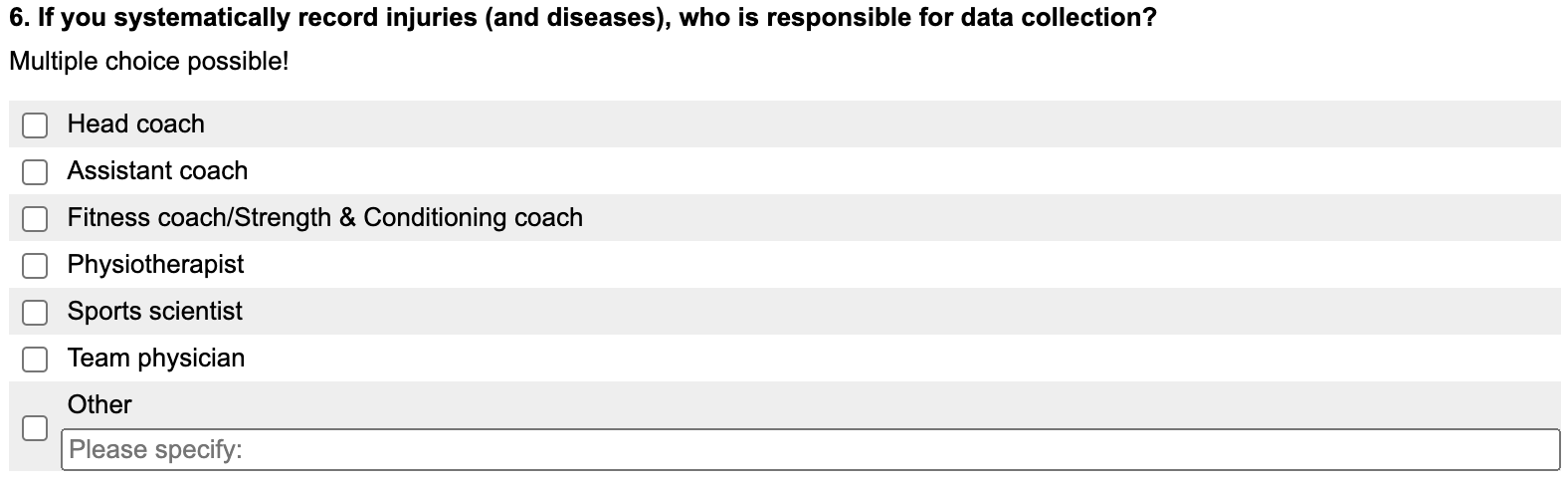

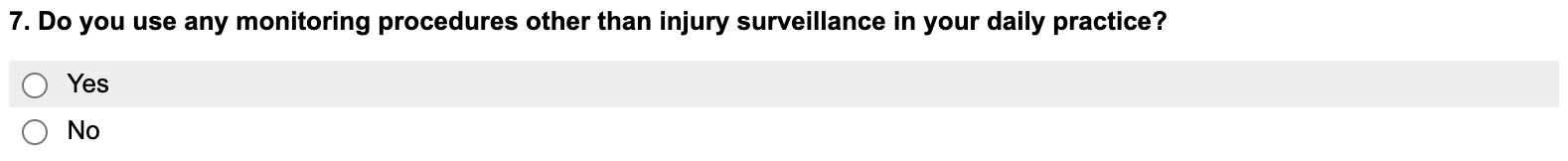

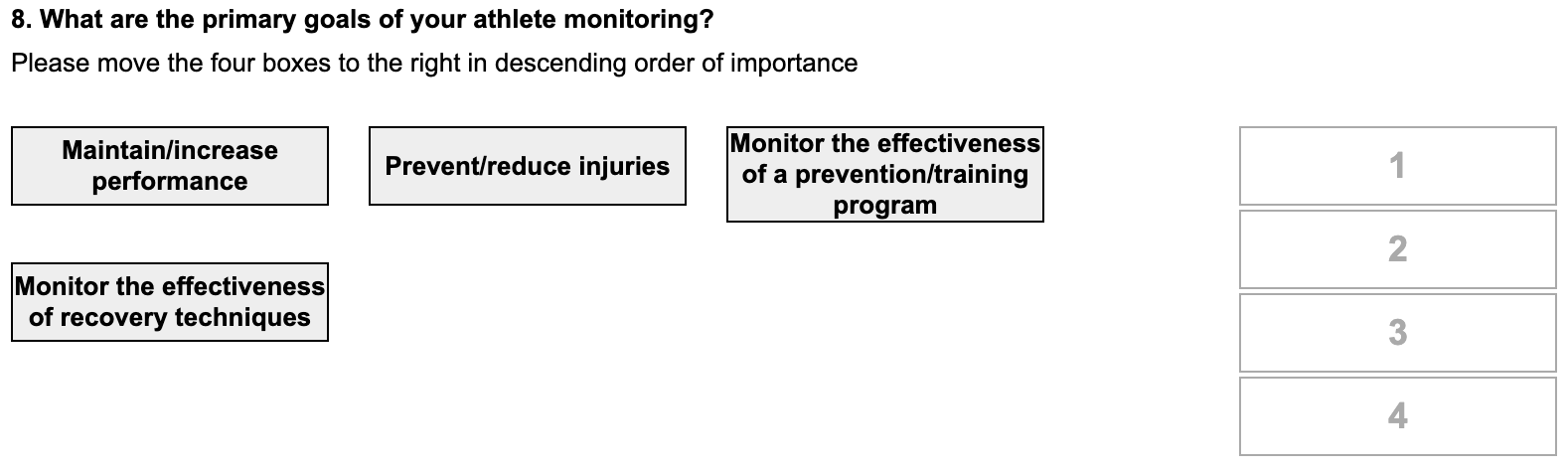

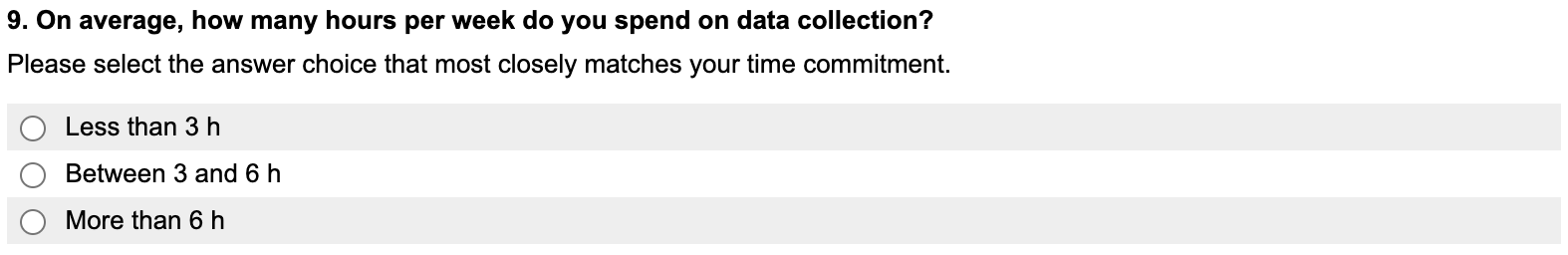


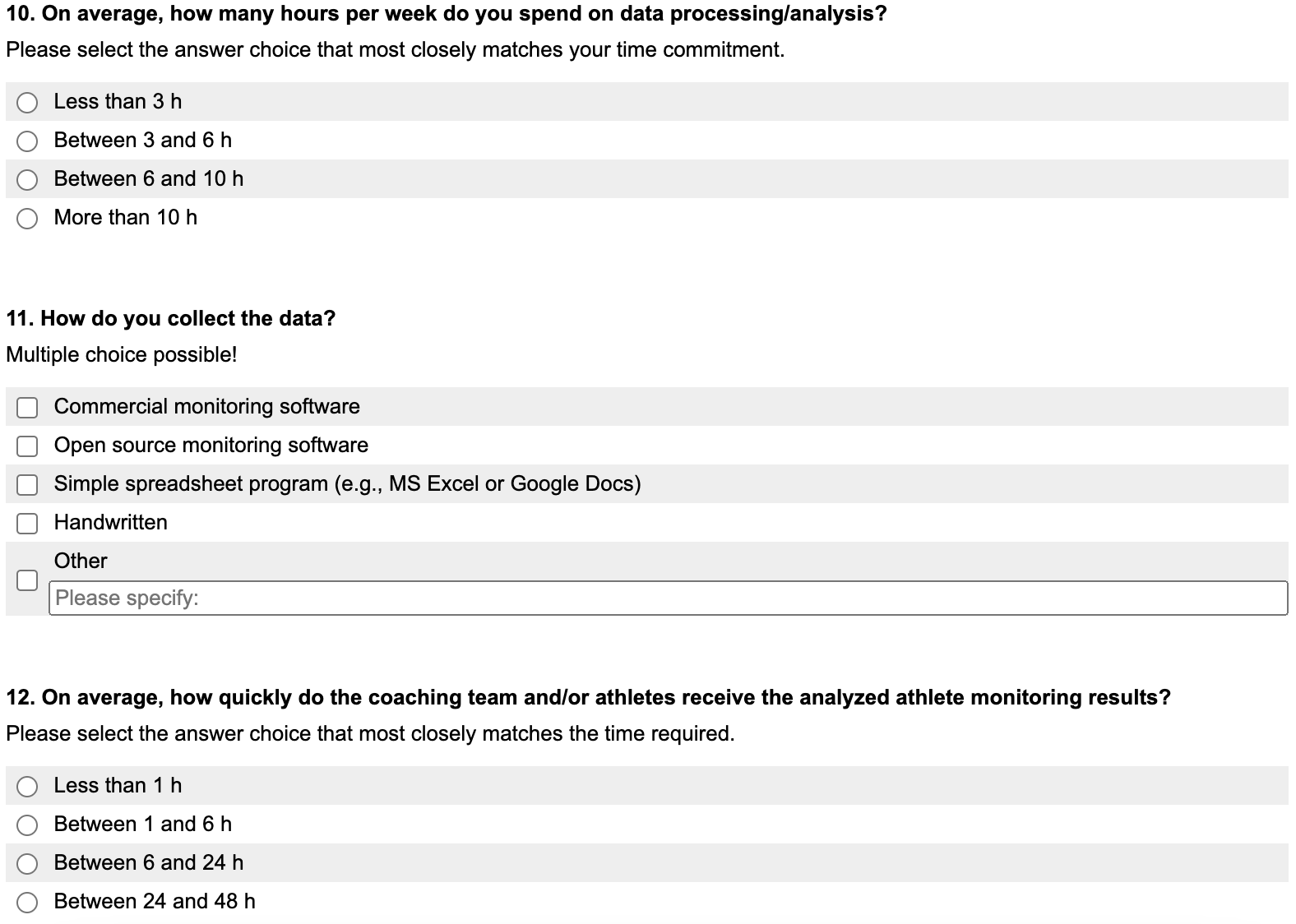

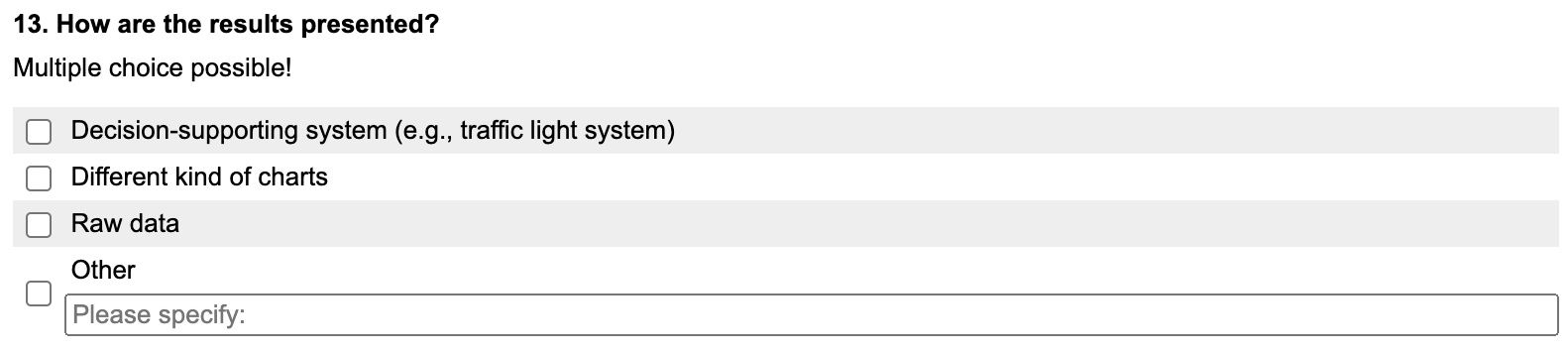

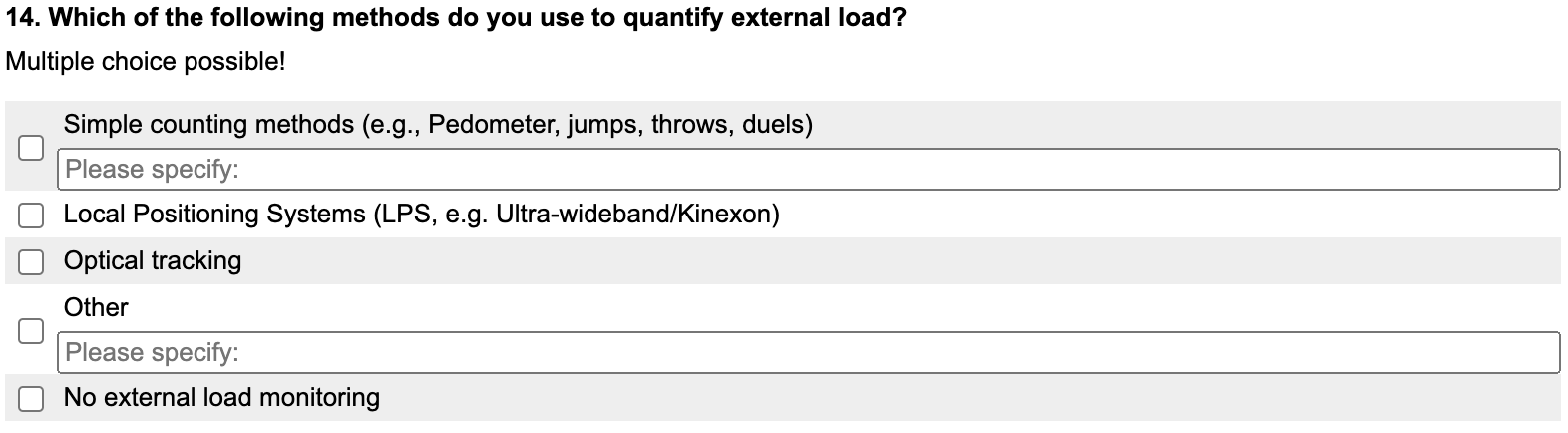

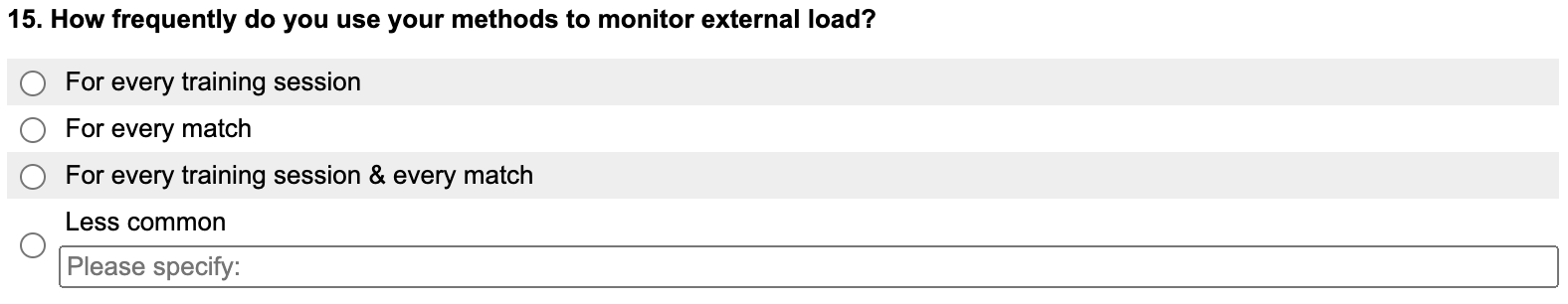


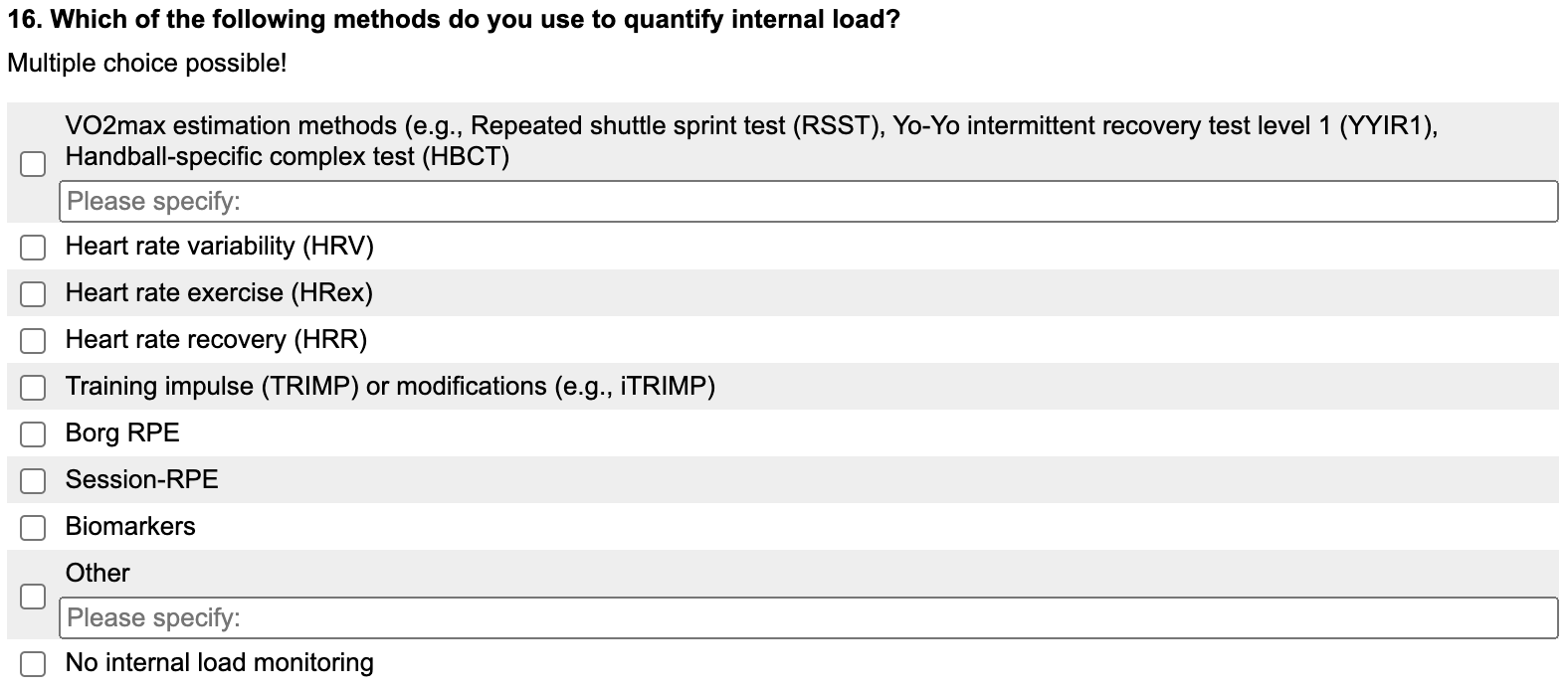

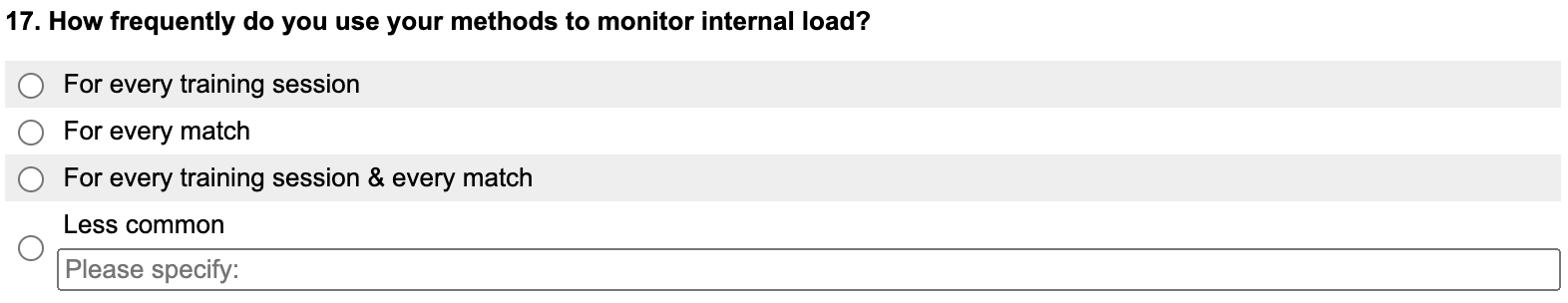

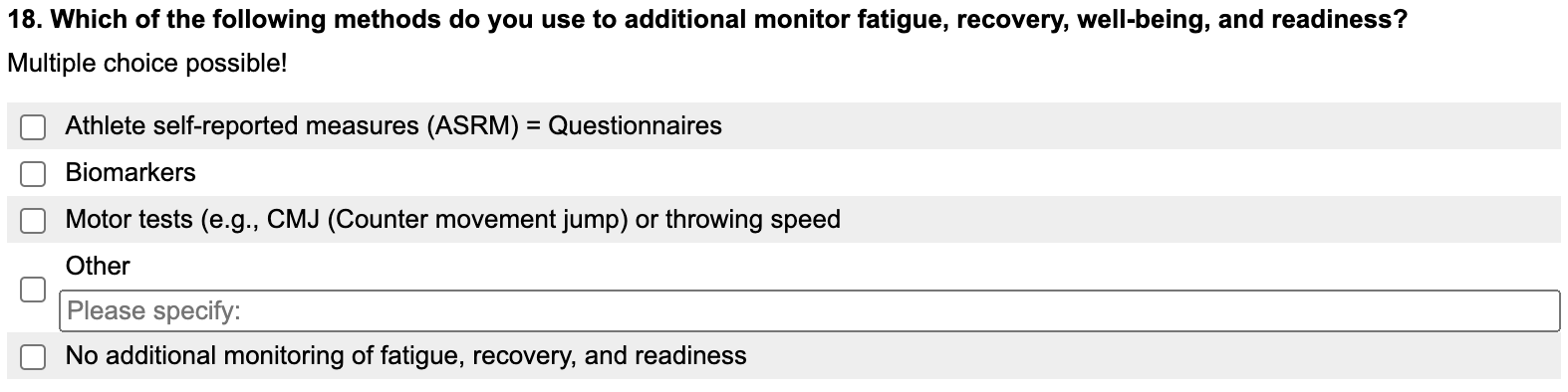

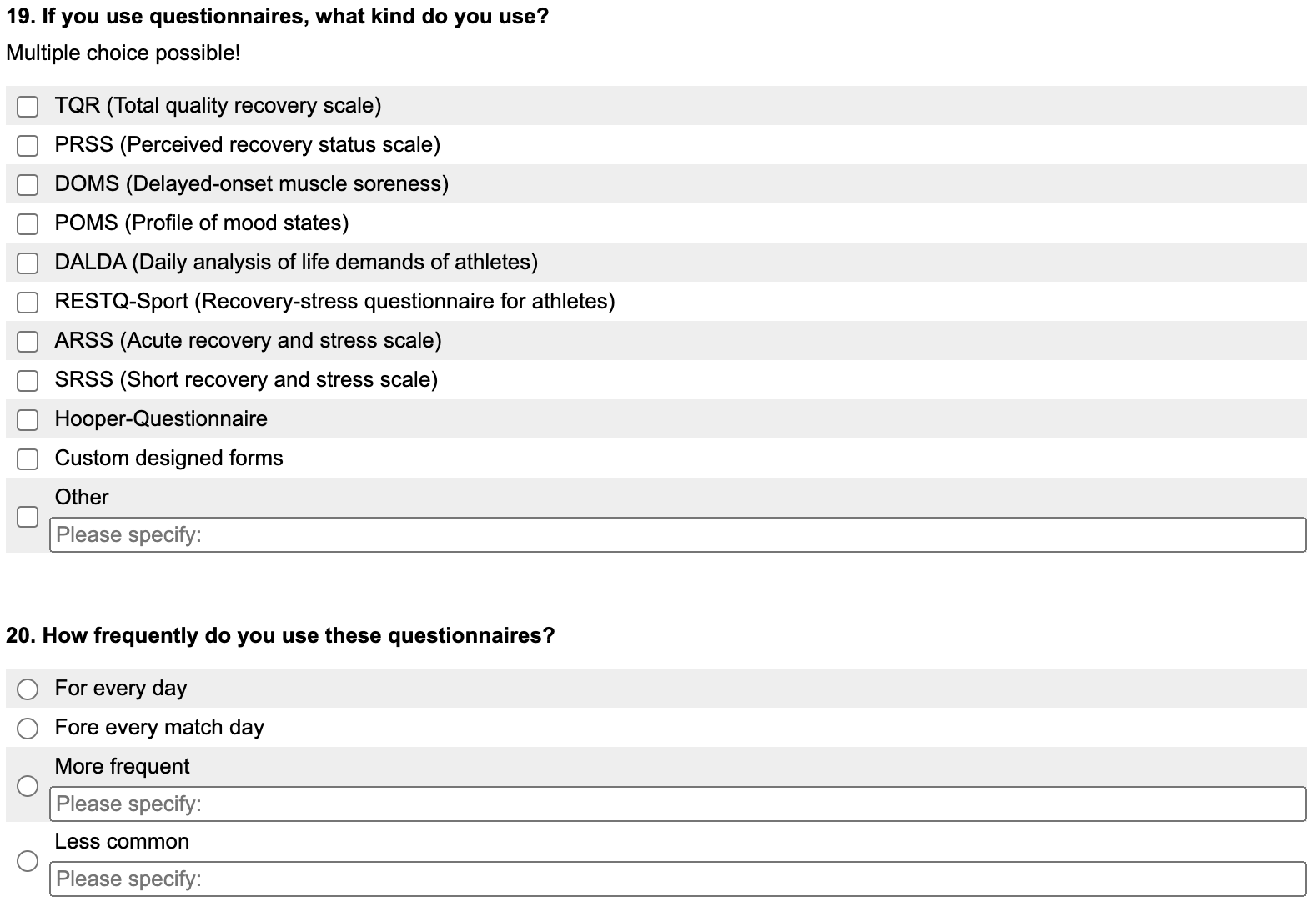


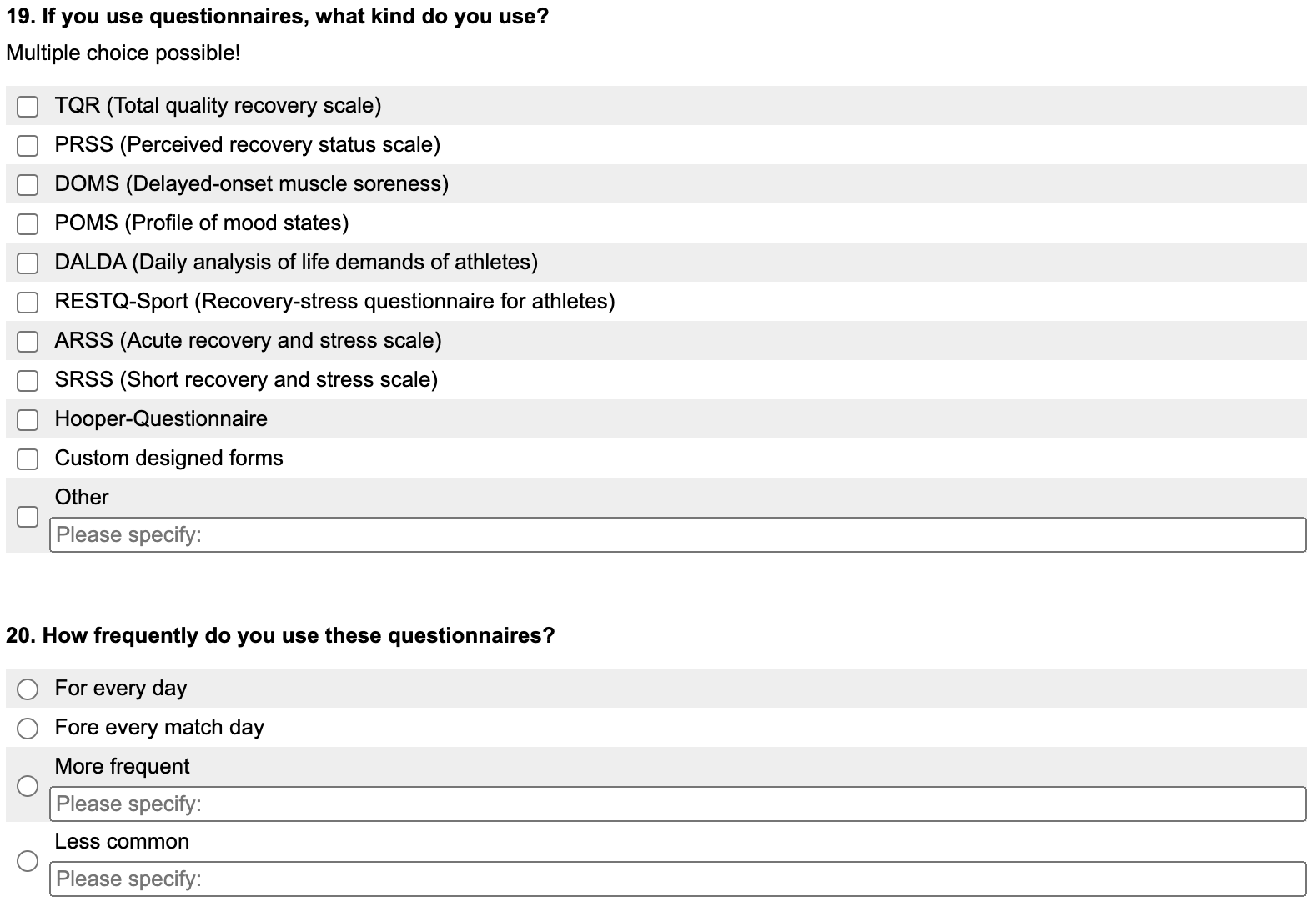

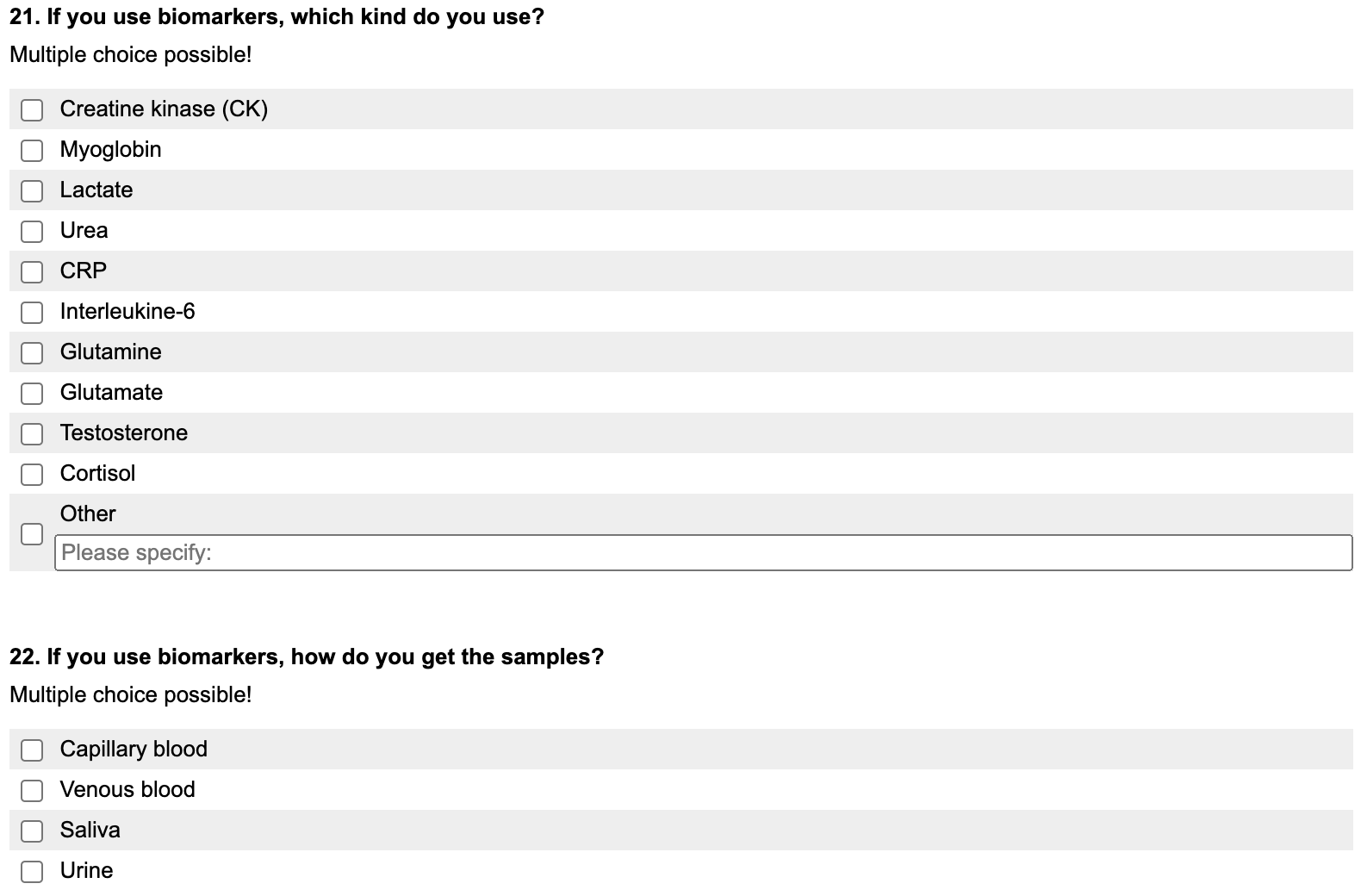

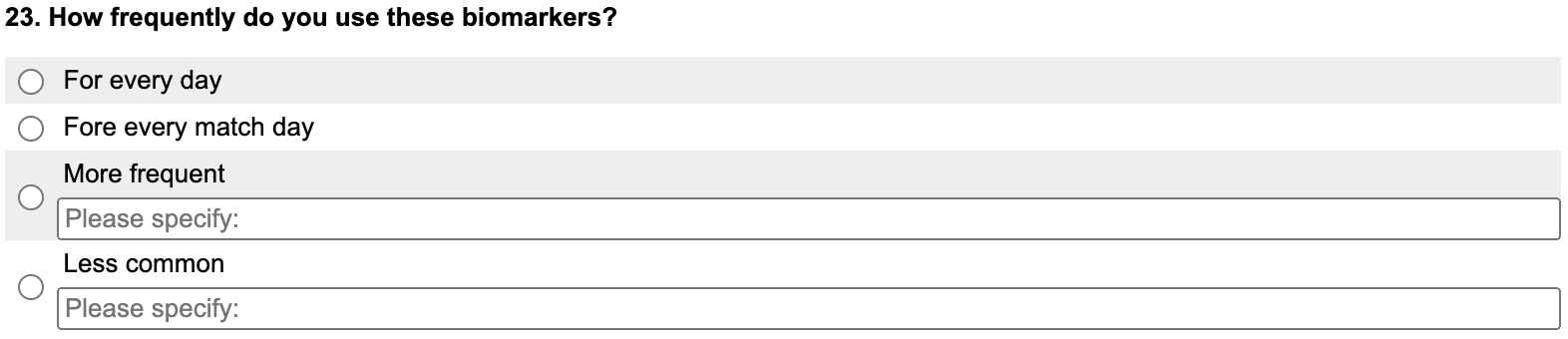

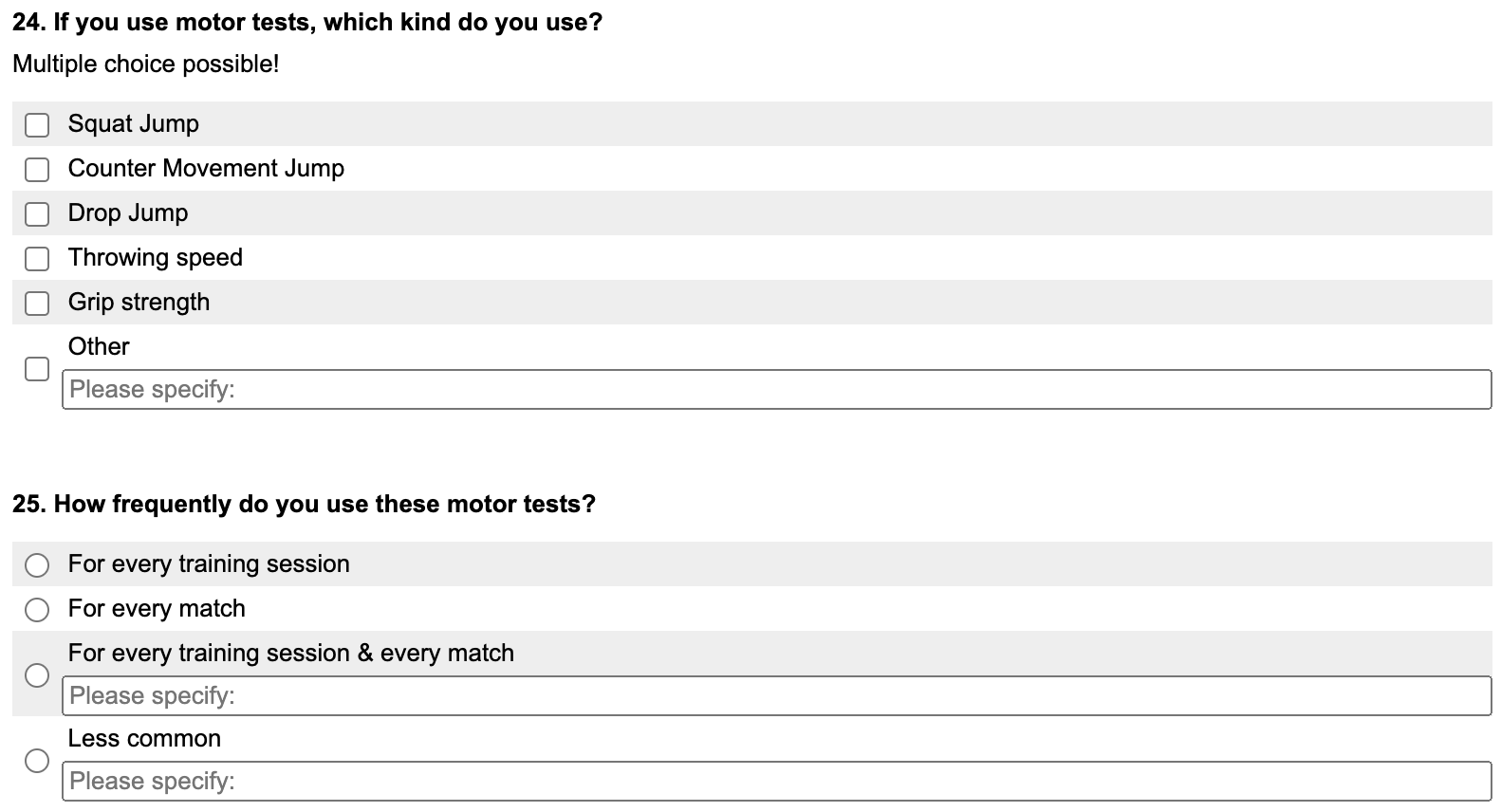


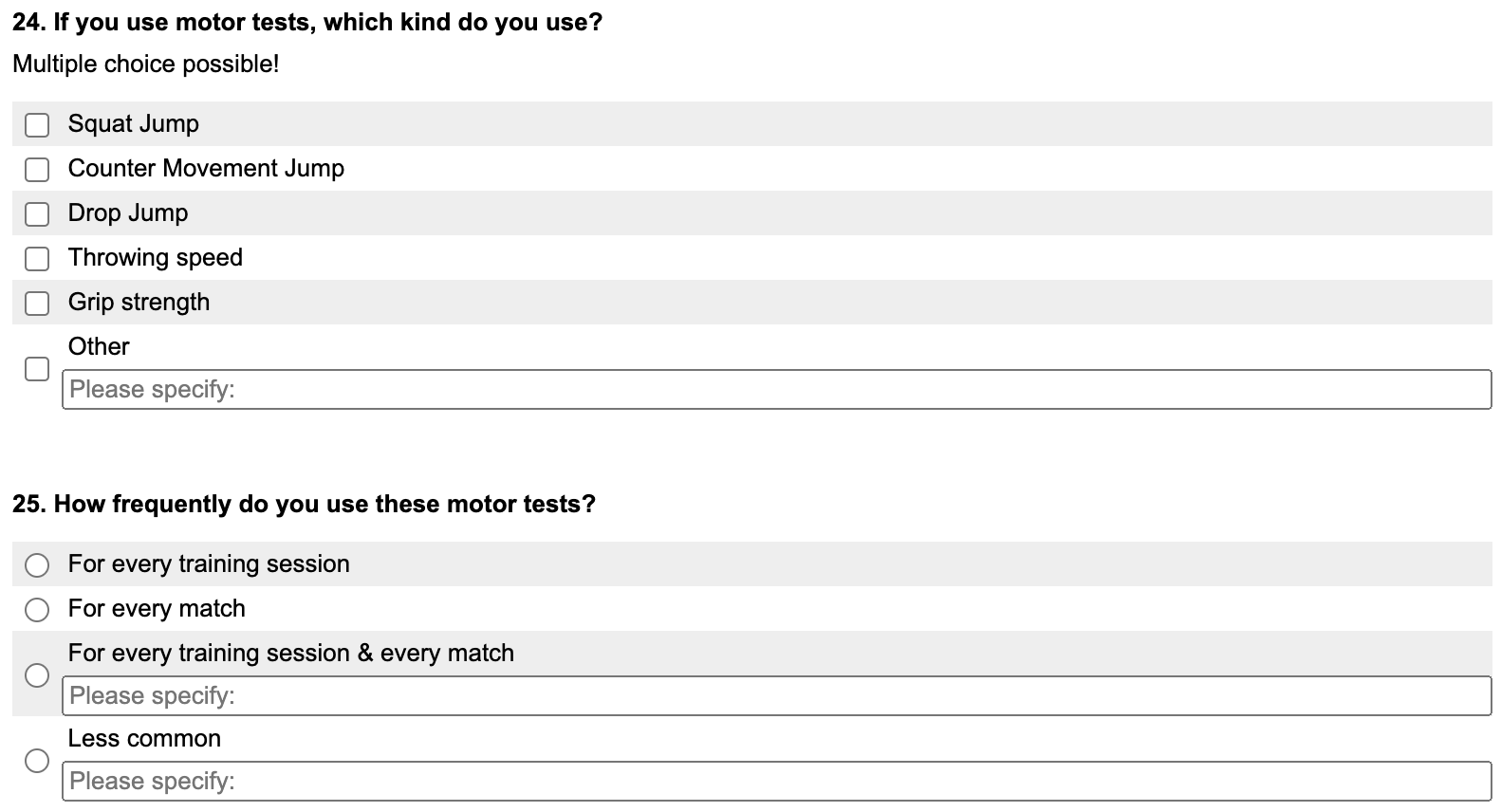

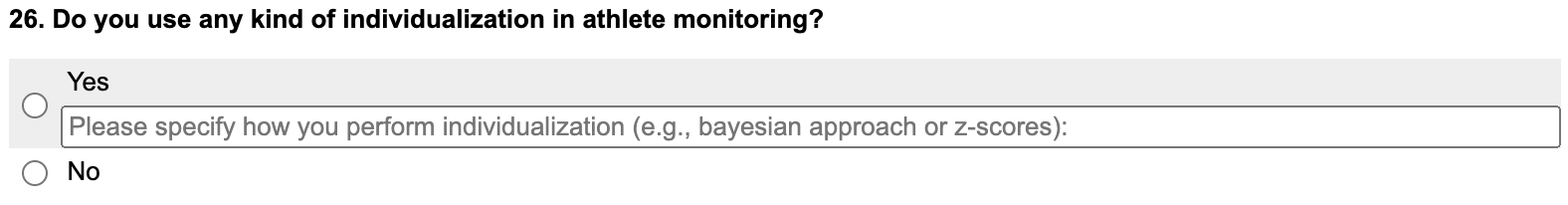

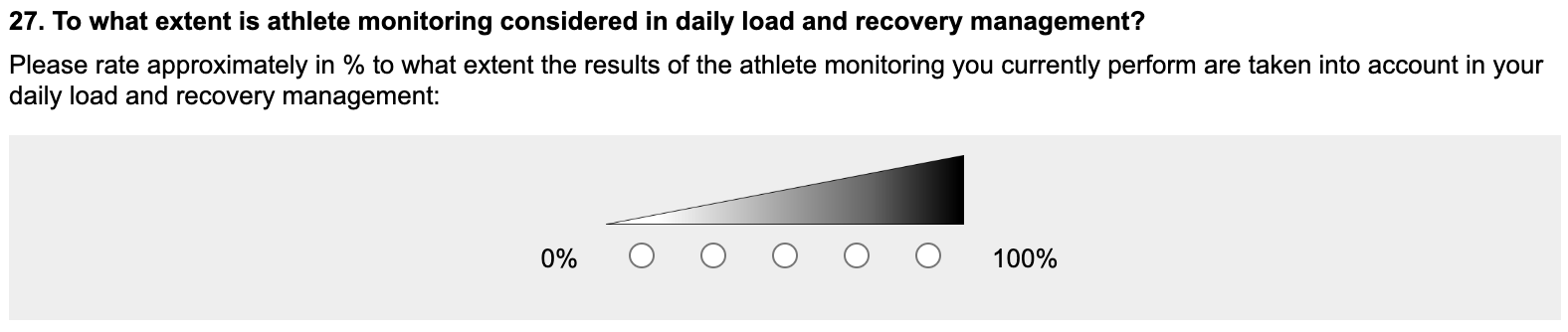

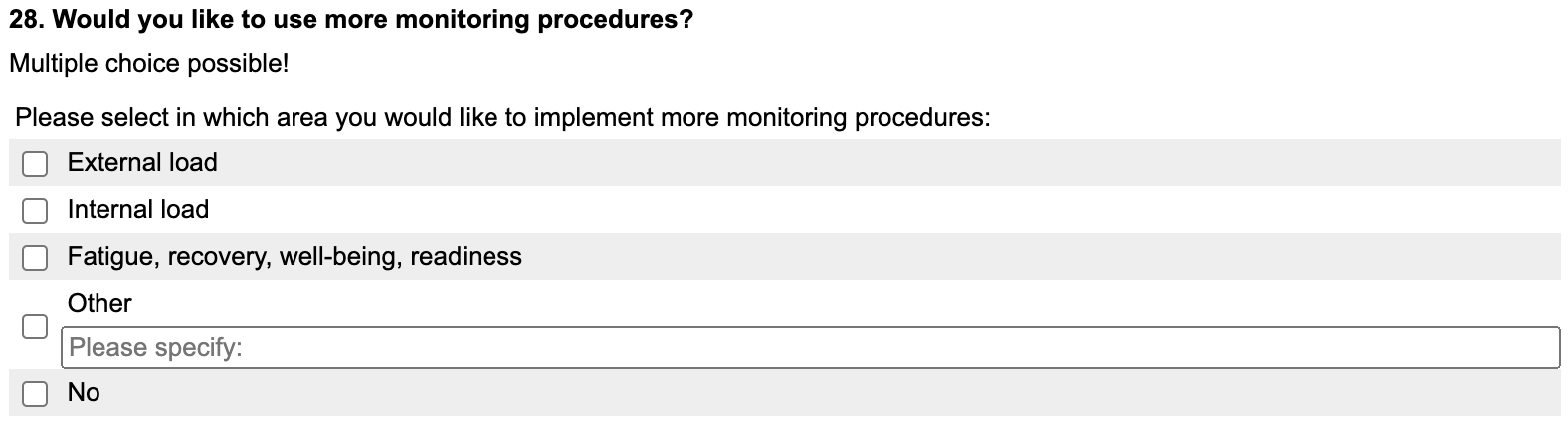

Supplement: Supplementary file 1 — Supplementary Material 1 [file 13102_2025_1177_MOESM1_ESM.docx]
